# The Prognostic and Therapeutic Potential of LRIG3 and Soluble LRIG3 in Glioblastoma

**DOI:** 10.3389/fonc.2019.00447

**Published:** 2019-06-06

**Authors:** Fangling Cheng, Po Zhang, Qungen Xiao, Youwei Li, Minhai Dong, Heping Wang, Dong Kuang, Yue He, Qiuhong Duan, Feng Mao, Baofeng Wang, Dongsheng Guo

**Affiliations:** ^1^Department of Neurosurgery, Tongji Hospital, Tongji Medical College, Huazhong University of Science and Technology, Wuhan, China; ^2^Chinese-German Lab of Molecular Neuro-oncology of Tongji Hospital, Huazhong University of Science and Technology, Wuhan, China; ^3^Institute of Pathology, Tongji Hospital, Tongji Medical College, Huazhong University of Science and Technology, Wuhan, China; ^4^Department of Pathology, School of Basic Medicine, Tongji Medical College, Huazhong University of Science and Technology, Wuhan, China; ^5^Department of Biochemistry and Molecular Biology, School of Basic Medicine, Huazhong University of Science and Technology, Wuhan, China

**Keywords:** glioma, glioblastoma, LRIG3, soluble LRIG3, MET/PI3K/Akt pathway, prognosis

## Abstract

Glioblastoma is a highly lethal type of primary brain tumor that exhibits unrestricted growth and aggressive invasion capabilities, leading to a dismal prognosis despite a multitude of therapies. Multiple alterations in the expression level of genes and/or proteins have been identified in glioblastomas, including the activation of oncogenes and/or silencing of tumor-suppressor genes. Nevertheless, there are still no effective targeted therapies associated with these changes. In this study, we investigated the expression of human leucine-rich repeats and immunoglobulin-like domains protein 3 (LRIG3) in human glioma specimens through immunohistochemical analysis. The results showed that LRIG3 was weakly expressed in high-grade gliomas (WHO [World Health Organization] grades III and IV) compared with that in low-grade gliomas (WHO grade II). Survival analysis of these patients with glioma indicated that LRIG3 is an important prognostic marker for better survival. Moreover, we confirmed the existence of soluble ectodomain of LRIG3 (sLRIG3) in the cell culture supernatant, serum, and in tumor cystic fluid of patients with glioma. Molecular mechanistic investigation demonstrated that both LRIG3 and sLRIG3 inhibit the growth and invasion capabilities of GL15, U87, and PriGBM cells and tumor xenografts in nude mice through regulating the MET/phosphatidylinositol 3-kinase/Akt signaling pathway. Enzyme-linked immunosorbent assay confirmed the positive correlation between serum sLRIG3 protein levels and overall survival time in patients with high-grade gliomas. Taken together, our data for the first time demonstrate the existence of sLRIG3 and that both LRIG3 and sLRIG3 are potent tumor suppressors, which could be used as prognostic markers for better overall survival and therapeutic agents for glioblastoma.

## Introduction

Glioblastoma (World Health Organization [WHO] grade IV) is a type of highly malignant brain tumor associated with high mortality, and has a median overall survival of 14.5–16.6 months (1). Even low-grade gliomas (LGGs; WHO grades I and II), most are inevitably recur and eventually progress to high-grade gliomas (HGGs; WHO grades III and IV [glioblastoma]) (2). Despite various salvage therapies (maximal safe resection, followed by radiochemotherapy and targeted therapy), once tumor progression occurs, the majority of patients succumb to the disease within 2 years of diagnosis (3). Multiple alterations in the expression level of genes and/or proteins have been identified in glioblastomas, including activation of oncogenes and/or silencing of tumor-suppressor genes (4). Many of them have been used as therapeutic targets and have their limitations in therapeutic applications (4). Therefore, further research is urgently needed to identify novel biomarkers for glioma, which can be used for screening high-risk patients, predicting prognosis, and application as a therapeutic target or agent.

The human leucine-rich repeats and immunoglobulin-like domains (LRIG) gene family was initially identified during the search for novel negative regulators of epidermal growth factor receptor (EGFR) (5). This gene family is composed of three paralogs, namely LRIG1, LRIG2, and LRIG3, encoding mono-transmembrane proteins with an ectodomain, a transmembrane domain, and a cytoplasmic tail (6). They share the same domain structure, but have different functions. LRIG1, negatively regulates several receptor tyrosine kinase (RTK) signaling pathways and is associated with a better prognosis in many cancers (7–10). LRIG2 acts as a tumor promoter, stimulating proliferation and inhibiting apoptosis of glioma cells (11, 12). Several studies have shown that soluble LRIG1 (sLRIG1) and soluble LRIG2 (sLRIG2) have similar functions as their corresponding full-length proteins (11, 13, 14). Meanwhile, the function of LRIG3 protein remains unclear. Previous studies demonstrated that LRIG3-deficient mice exhibit craniofacial and inner ear defects (15), cardiac hypertrophy and decreased high-density lipoprotein cholesterol (16). Another study showed that LRIG3 could stabilize the ErbB receptor and function as a tumor promoter in HEK293T cell line (17). In contrast, several other studies demonstrated that LRIG3 inhibited the proliferation, apoptosis, and invasion of glioma cells by negatively regulating the EGFR signaling pathway (18, 19). Because of the contradiction in LRIG3 protein function and the fact that the presence and function of soluble LRIG3 (sLRIG3) in gliomas remain unknown, it is essential to further investigate the characteristics of LRIG3 in gliomas.

A previous analysis of glioma samples showed that approximately 86% harbor at least one genetic event in the core RTK/PI3K pathway (20). The amplification and activation of EGFR, platelet derived growth factor receptor α (PDGFRα), and mesenchymal-epithelial transition factor (MET) promote the proliferation and invasion of glioma cells and are correlated with recurrence and therapeutic resistance (21–24). Of note, MET and its ligand hepatocyte growth factor (HGF) play a critical role in the proliferation, survival, migration, invasion, angiogenesis, stem cell characteristics, and therapeutic resistance and recurrence of glioblastomas (25–30). Both higher MET expression and MET gene gain are associated with poorer median survival for glioblastoma or diffuse astrocytoma (31–33). Since many studies on LRIG gene family members are associated with the regulation of RTKs, it is worthwhile to further elucidate whether LRIG3 regulates the MET signaling pathway in glioblastoma.

In the current study, expression levels of LRIG3 in the glioma tissue samples and sLRIG3 in patients' serum and glioma cystic fluid were examined. The functions of LRIG3 and sLRIG3 were evaluated both *in vitro* and *in vivo*. Our results suggested that LRIG3 and sLRIG3 may represent novel prognostic markers for patients with glioblastoma or HGG and could be used as potential therapeutic agents in future treatment strategies.

## Materials and Methods

### Ethics Statement and Patient Follow-Up

All the samples were obtained from the Department of Neurosurgery, Tongji Hospital, Huazhong University of Science and Technology (Wuhan, China) after written consent and according to a protocol approved by the Research Ethics Committee of Tongji Hospital, Tongji Medical College, Huazhong University of Science and Technology (serial no. TJ-IBR20181111). Patient data and samples were handled according to the ethical and legal standards adopted by the Declaration of Helsinki (2013). Follow-up information of all eligible patients was updated every 3 months via telephone interviews or outpatient reviews. Overall survival (OS) was calculated from the date of the initial surgery to the date of death or 30 months after surgery.

### Tumor Specimens and Control Specimen

All tumor tissue samples were collected from September 2014 to December 2017. Tumor specimens for immunohistochemistry (IHC) staining were surgically removed from 65 patients (32 males and 33 females), while specimens for western blotting were collected from 16 patients (9 males and 7 females). One normal brain tissue sample was obtained from decompression surgery of a patient with traumatic brain injury. Histopathological diagnosis was made by neuropathologists based on the World Health Organization (WHO) classification. Formalin-fixed, paraffin-embedded glioma samples and frozen glioma samples were separately stored at room temperature and −80°C. The characteristics of these patients with glioma are summarized in Table 1 and Table S1.

**Table 1:** Characteristics of glioma patients with IHC staining.

**Characteristics**	**WHO Grade**			**Total**
	**II**	**III**	**IV**	
All patients	20	17	28	65
**SEX**				
Male	6	8	18	32
Female	14	9	10	33
**AGE**				
Median	43	44	55	47
Range	30–63	14–68	16–73	16–73
**LOCATION**				
Left	10	8	15	33
Right	9	9	12	30
Both	1	0	1	2
**INVOLVED BRAIN LOBES**				
Frontal lobe	14	10	14	38
Temporal lobe	8	17	14	39
Parietal lobe	4	5	6	15
Occipital lobe	0	0	2	2
Insular & basal ganglia	3	3	4	10
Multiple lobes	8	7	10	25
**HISTOPATHOLOGIC DIAGNOSIS**				
Astrocytoma	14	14	0	28
Oligodendroglioma	3	1	0	4
Oligoastrocytoma	3	2	0	5
Glioblastoma	0	0	28	28
**Median overall survival (months)**	–	17	14.5	20

*These samples were collected from September 2014 to September 2015*.

### Immunohistochemistry Staining

The tumor samples were fixed in 4% paraformaldehyde immediately after resection, then dehydrated with ethanol gradient, permeabilized with xylene and paraffin embedded (*n* = 65). Immunohistochemistry tissue sections (4 μm thick) were generated from paraffin-embedded tissues and then stained with primary rabbit anti-LRIG3 antibody (Abcam; Cat# ab197989) as follows. The antigen retrieval was carried out in a microwave with citrate buffer (pH 6.0) and the inactivation of endogenous peroxidase was performed in 3% H_2_O_2_. Three percentage bovine serum albumin (BSA) was added to cover the tissue evenly to block the non-specific antigen at room temperature for 30 min. Then primary antibodies were added to each slide at appropriate dilutions and incubated in 4°C overnight. The next day, the sections were incubated with biotin-labeled secondary antibodies for 50 min. The final signals were developed using the 3,3'-diaminobenzidine substrate (DAB) (RD, America). The sections were analyzed by optical microscopy after counterstaining with hematoxylin.

LRIG3 staining was semi-quantitatively stratified and scored by percentage positivity of tumor cells (0–10% = 1, 11%−30% = 2, 31%−50% = 3, and 51%−100% = 4) and staining intensity (none = 0, weak = 1; moderate = 2; intense = 3) according to Yuan et al. (34). Finally, an overall score (0–12) was calculated for each case by multiplying the percentage score with intensity score.

Series continuous tissue sections of representative gliomas with different grade were stained with GFAP (Cell Signaling Technology, Cat# 80788), Iba-1 (Abcam, Cat# ab5076), CD163 (Abcam, Cat# ab182422), and CD31 (Proteintech, Cat# 11265-1-AP).

The IHC staining for tumor xenografts was performed as described above with the following antibodies: mouse anti-FLAG (Sigma-Aldrich, Cat# F1804), rabbit anti-phospho-MET (Cell Signaling Technology, Cat# 3077s), rabbit anti-phospho-Akt (Cell Signaling Technology, Cat# 4058) and rabbit-anti-Ki-67 (Servicebio, Cat# GB13030-M-1).

### Processing of Serum and Glioma Cystic Fluid

Blood samples (3 ml) from glioma patients (*n* = 50) and healthy controls (blood samples from healthy volunteers; *n* = 14) were collected from the Tongji Hospital, Tongji Medical College, Huazhong University of Science and Technology between December 2014 and December 2015. All blood samples were allowed to clot for 2–3 h at room temperature (about 28°C) and serum was recovered after centrifugation (3000 × *g*, 5 min) and stored at −80°C. Cystic fluid from cystic glioma lesions was obtained during tumor resection surgery by aspiration into a sterile syringe, which was emptied into a polypropylene tube. These fluid samples were clear, light yellowish, which were clearly cystic fluid and not necrotic tumor material. These samples were stored at −80°C after centrifugation to discard the precipitated cells. The patient characteristics of blood serum and tumor cystic fluid samples were summarized in Table 2 and Table S2.

**Table 2:** Characteristics of the patients and normal people with serum samples for sLRIG3 detection.

	**Normal**	**I**	**II**	**III**	**IV**
All patients	14	6	11	6	27
**SEX**					
Male	4	3	3	2	19
Female	10	3	8	4	8
**AGE**					
Median	51	7.5	43	58.5	50
Range	24–66	1.5–16	23–54	45–64	15–72
**SURGICAL TREATMENT**					
Resection	–	6	11	6	25
Biopsy	–	0	0	0	1
Non	–	0	0	0	1
**Median overall survival (months)**	–	–	–	18	15
sLRIG3 (ng/ml)^*^	1.01 ± 0.27	1.47 ± 0.77	2.05 ± 0.74	3.67 ± 0.95	3.95 ± 0.91

*These samples were collected from December 2014 to December 2015*.

* *represents mean ± SEM*.

### Expression Constructs and Stable Transduction

The inducible pLVX-TRE3G-LRIG3 expression vector was donated by Dr. Håkan Hedman (Umeå University Hospital, Sweden) to generate stable transduced glioma cell lines. Plasmids pLVX-Puro-3 × FLAG-LRIG3 (LRIG3-full) and pLVX-Puro-3 × FLAG-LRIG3ecto (LRIG3-ecto) were generated from the pEGFP-LRIG3 plasmid (gifted by Håkan Hedman) as previously described (14). Glioma cell lines expressing full-length LRIG3 or the ectodomain of LRIG3 were generated using the Lenti-X Lentiviral Expression System (Clontech, Mountain View, CA), according to the manufacturer's instructions.

### Cell Lines and Cell Culture

The cell lines U87 and U251 (purchased from the American Type Culture Collection), GL15 (donated by Dr. Håkan Hedman, Umeå University Hospital, Sweden), U373 (obtained from the Cancer Center of Union Hospital, Tongji Medical College), and HS683 and A172 (donated by Prof. Qiuhong Duan, Tongji Medical College) were cultured in DMEM (Dulbecco's Modified Eagle's medium)/high glucose supplemented with 10% fetal bovine serum ([FBS] 04-001; Biolnd), 100 U/ml penicillin, and 0.1 mg/ml streptomycin in a humidified incubator with 5% CO_2_ at 37°C.

The patient-derived adherent cell line, named PriGBM in this study, was cultured and established in our laboratory in 2016. This cell line has been further identified for short tandem repeat (STR) analysis, bacterial and mycoplasma contamination in Bio-research Innovation Center, Suzhou Institute of Biochemistry and Cell Biology (BRICS), Chinese Academy of Sciences. It was also cultured under the same conditions as GL15 and other cell lines.

Cells transduced with the pLVX-TRE3G-LRIG3 vector were cultured in DMEM supplemented with 10% (v/v) FBS (tetracycline-free; 04-005; Biolnd), without penicillin-streptomycin. When indicated, LRIG3 expression in these cells was induced by the addition of 1 μg/ml doxycycline (Dox; Clontech) to the cell culture medium (35).

Cells stably transduced with the control (pLVX-Puro), pLVX-Puro-3 × FLAG-LRIG3-full, or pLVX-Puro-3 × FLAG-LRIG3-ecto expression vectors were cultured as non-transduced cells. For the detection of recombinant human HGF (rhHGF, PeproTech 100-39) induced phosphorylation of MET, Akt, and mTOR, 20 ng/ml rhHGF was added into the culture medium and incubated for 15 min, and then the cells were lysed for western blotting.

### Western Blotting and Antibodies

Human glioma tissue samples were immersed in RIPA buffer (Beyotime Biotech, China), disrupted by ultra-sonication, lysed, and centrifuged at 12,000 × *g* for 15 min. The supernatants were then collected for western blotting. The lysates of the cultured cells were harvested as previously described and equal amounts of total protein (10–50 μg) were loaded for western blotting (36).

All protein samples were separated by sodium dodecyl sulfate polyacrylamide gel electrophoresis (SDS-PAGE) on 8 or 10% gels, transferred on to nitrocellulose membranes (Millipore, Burlington, MA), and detected using the WesternBright ECL kit (Advansta, Cat# K-12045-D50). The amount of proteins loaded and antibody dilutions used were optimized in our laboratory. The following primary antibodies were used: mouse anti-FLAG (Sigma, Cat# F1804), goat anti-LRIG3 (R&D Systems, Cat# AF3495, recognizes the ectodomain of LRIG3 protein, Arg28-Thr807), rabbit anti-EGFR (Abcam, Cat# ab131498), rabbit anti-phospho-EGFR (Cell Signaling Technology, Cat# 3777s), rabbit anti-PDGFRα (Cell Signaling Technology, Cat# 3174S), rabbit anti-phospho-PDGFRα (Cell Signaling Technology, Cat# 2992), rabbit anti-MET (Cell Signaling Technology, Cat# 8198), rabbit anti-phospho-MET (Cell Signaling Technology, Cat# 3077), rabbit anti-Akt (Cell Signaling Technology, Cat# 9272), rabbit anti-phospho-Akt (Cell Signaling Technology, Cat# 4058), rabbit anti-mTOR (Cell Signaling Technology, Cat# 2972), rabbit anti-phospho-mTOR (Cell Signaling Technology, Cat# 5536), rabbit anti-cyclinD1 (Cell Signaling Technology, Cat# 2978), and mouse anti-α-Tubulin (Proteintech, Cat# 66031-1). Secondary antibodies (goat anti-mouse, goat anti-rabbit, and rabbit anti-goat antibodies) were all purchased from Earth-Ox Life Sciences (Millbrae, CA).

### Cell Proliferation Assay

Cells were seeded into a 96-well plate with 2 × 10^3^ cells (4 × 10^3^ cells for A172) per well in 100 μl suspension in triplicate and maintained in complete culture medium for 5 days (three days for A172). Cell proliferation rates were measured using the cell counting kit-8 (CCK-8; KJ800; Dojindo Laboratories) according to the manufacturer's instructions.

### Detection of sLRIG3

Cells stably transduced with pLVX-TRE3G-LRIG3 were cultured in DMEM (with 10% FBS) with or without Dox (1 μg/ml) for 48 h, and then maintained in DMEM (without FBS) with or without Dox for another 24 h. The culture medium was harvested separately, concentrated and subjected to western blotting for the detection of sLRIG3 as previously described (11). SLRIG3 in serum samples and tumor cystic fluid was detected by western blotting at a dilution of 1:15. Further quantitative analysis of sLRIG3 in serum samples was performed using an enzyme-linked immunosorbent assay kit (ELISA; ELH-LRIG3-1; RayBiotech), according to the manufacturer's instructions.

### Immunofluorescence Assay

GL15 and PriGBM cells stably transduced with empty control vector pLVX-Puro, and pLVX-Puro-3 × FLAG-LRIG3-full or pLVX-Puro-3 × FLAG-LRIG3-ecto expression vectors were seeded in 6-well culture plates. After 48 h of incubation, the cells were fixed with 4% paraformaldehyde for 1 h at 4°C, permeabilized with 0.25% Triton X-100 for 5 min, blocked with10% BSA for 1 h, and then incubated with anti-FLAG antibody (Sigma, Cat# F1804), overnight at 4°C at a dilution of 1:1000. After washing with PBS, cells were incubated with FITC-labeled secondary antibody (1:100; Proteintech) and subjected to immunofluorescence microscopy using appropriate filters.

### Soft Agar Assay

For the soft agar assay, 0.5% basal medium Eagle (BME) agar containing 10% FBS, 2 mM L-glutamine, and 25 μg/ml gentamicin, was added to 6-well plates (3 ml/well), with three wells per group. The plates were then placed in the incubator for 1 h for coagulation of the semi-solid medium. Subsequently, glioma cells were resuspended at a concentration of 8 × 10^3^/ml in 0.33% BME agar containing 10% FBS, 2 mM L-glutamine, and 25 μg/ml gentamicin and were seeded at 1 ml/well. The cultures were maintained at 37°C in a 5% CO_2_ incubator for 14 days, and then the cell colonies were observed by microscopy.

### Invasion and Migration Assays

The *in vitro* invasive capability of GL15, U87 and PriGBM cells was measured using the transwell invasion assays. Briefly, 50 μl of diluted Matrigel solution (1:4; Corning, Cat# 356234) was added to the upper chambers of the Transwell inserts (6.5 mm, 8-μm pore size; BD Biosciences). The inserts were incubated for 1 h at 37°C for gelling. Cells were added at a density of 5 × 10^4^ cells in 100 μl of medium with 2% FBS to the upper chambers, while the lower chambers were filled with 600 μl of DMEM containing 10% FBS. The Transwell inserts were then incubated for 24 h and the subsequent steps were performed as previously described (37).

The migration ability of cells in different groups was examined by *in vitro* wound-healing assay. Cells were seeded and were cultured into the 6-well plates untail they reached a 80–90% confluence and then cultured with serum-free DMEM for 24 h. After that, similar size scratches were introduced into the monolayer by a sterile pipette tip. The monolayer cells were rinsed with PBS to remove detached cells, and then replaced and cultured with serum-free DMEM. To analyze the cell migration, the wounded areas were photographed at the indicated 24 h point and then calculated the vacant area of each photo using Photoshop CS5. Percentage of wound healing was calculated as following: [1—(empty area 24h/empty area 0 h)] × 100%.

### SiRNA-mediated Gene Silencing

Three siRNA duplexes, si-1-LRIG3 (target sequence: 5′-GCTGGACCATAACAACCTA-3′), si-2-LRIG3 (target sequence: 5′-GGACCAGTCTCGGCAAATA-3′), and si-3-LRIG3 (target sequence: 5′-GGATCCGTTCTATTACTAA-3′), were designed to target different coding regions of the human *LRIG3* mRNA sequence (GeneBank accession no. NM_153377.4 and NM_001136051.2) and purchased from RiboBio (Guangzhou, China). A172 cells were transfected with these siRNA and control siRNA using Lipofectamine RNAiMAX transfection reagent (Invitrogen Life Technologies, Carlsbad, CA, USA), according to the manufacturer's instructions.

### *In vivo* Tumor Model

Male BALB/c athymic nude mice (4–5 weeks of age) were purchased from Hunan Silaike Jingda Laboratory Animal Co. Ltd. (Changsha, China) and housed under specific pathogen-free conditions in a temperature- and humidity-controlled environment. GL15 cells stably expressing full-length LRIG3 (LRIG3-full) or LRIG3 ectodomain (sLRIG3) proteins, as well as control cells, were harvested and injected subcutaneously into the right axilla of each mouse (2.5 × 10^6^ cells/100 μl/mouse, 5 mice/group). Tumor volume was determined by caliper measurements every 10 days and calculated using the following formula: volume = width^2^ × length/2, where length is the longest diameter and width is the shortest diameter perpendicular to the length (38). Sixty days after inoculation, all mice were euthanized and the tumors were surgically harvested, measured, fixed in 4% paraformaldehyde overnight, embedded in paraffin, and analyzed by IHC staining. All animal experiments were conducted in accordance with the Institutional Animal Care and Use Committee guidelines and the animal protocol used in this study was approved by the Ethical Committee of Tongji Hospital, Tongji Medical College, Huazhong University of Science and Technology (serial no. TJ-A20161206).

### Statistical Analysis

Data was graphed using GraphPad Prism 5 Software (GraphPad Software Inc., La Jolla, CA) and the statistical analyses were performed using SPSS 16.0 software (IBM, Armonk, NY). Kaplan–Meier method was used to construct survival curves with a log-rank test. For comparison of experimental groups (>2 groups), multiple comparisons were performed using the one-way analysis of variance (one-way ANOVA) followed by Bonferroni *post-hoc* test, while for comparison of two groups, we used Student's *t*-test. For the data from our laboratory with repeated experiments, mean values were reported together with SD. For the data from clinical patients, which holds more samples of the patient population, we used mean ± SEM. Pearson correlation analysis was performed to analyze the correlation between the expression levels of LRIG3 protein and the activation levels of MET and Akt proteins in the 16 glioma tissue samples. Differences with *p* < 0.05 were considered statically significant.

## Results

### LRIG3 Expression Correlates With Prognosis in Patients With Glioma

Our previous study demonstrated that LRIG3 could modulate the proliferation, apoptosis, and invasion of glioma cells and functions as a tumor suppressor by attenuating the EGFR signaling pathway (19). To interrogate the clinical significance of LRIG3 in gliomas, tumor samples from 65 patients (20 cases of grade II, 17 cases of grade III, and 28 cases of grade IV) were collected for LRIG3 IHC staining to analyze the correlation between LRIG3 protein expression levels and overall survival.

**Figure 1:**
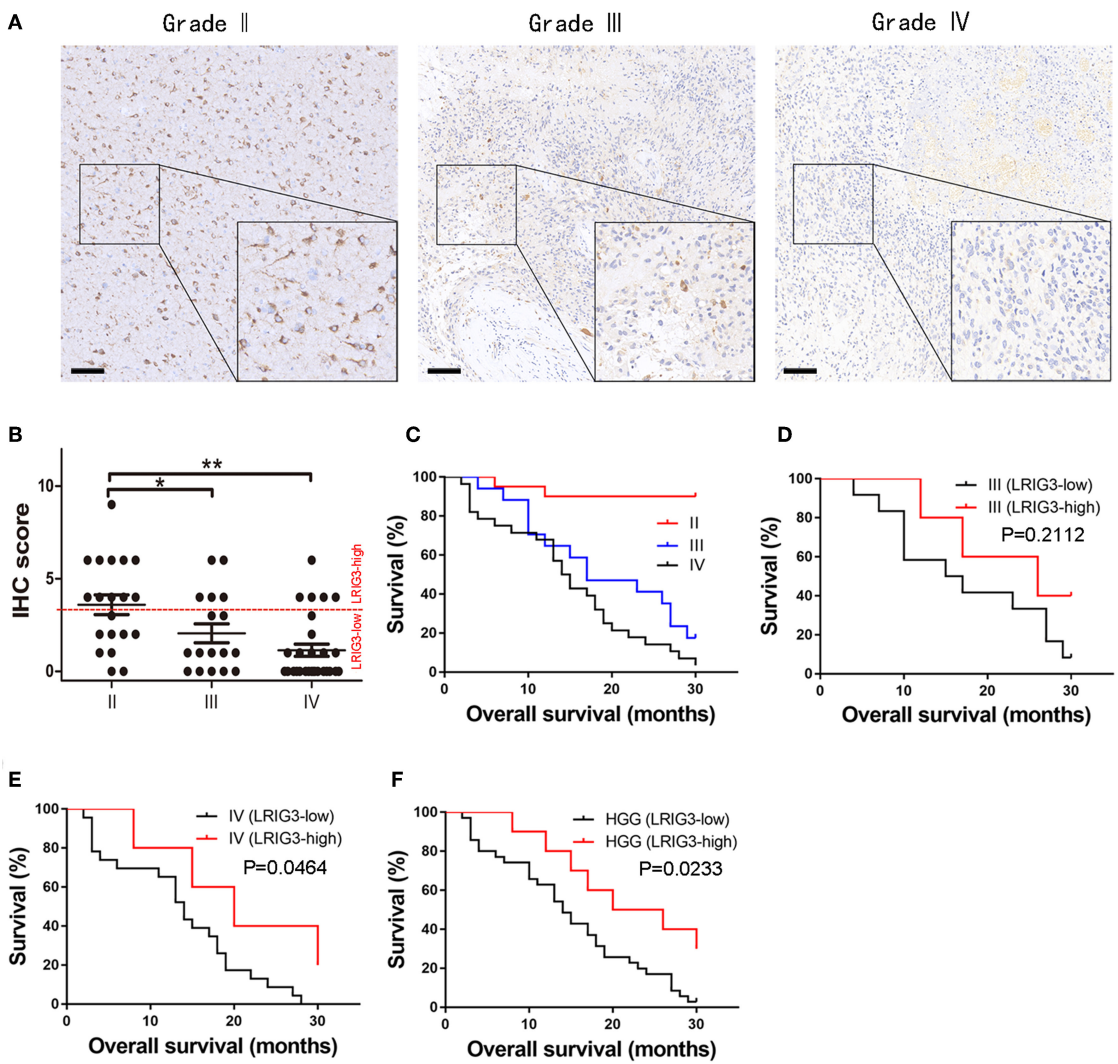
LRIG3 expression levels are correlated with the prognosis of glioma patients. **(A)** Representative IHC staining of LRIG3 protein expression in human glioma tissue sections of different WHO grades. Scale bars, 100 μm. **(B)** Immunostaining scores of LRIG3 in the 65 paraffin-embedded sections of different grade glioma samples. Data represent the mean ± SEM; ^*^p < 0.05; ^**^p < 0.01; one-way ANOVA. **(C)** Kaplan–Meier survival curves showing overall survival of the 65 patients with grades I, II, and IV gliomas. **(D)** Kaplan–Meier survival curves of grade III glioma patients with different LRIG3 expression levels. **(E)** Kaplan–Meier survival curves of grade IV glioma patients with different LRIG3 expression levels. **(F)** Kaplan–Meier survival curves of HGG patients with different LRIG3 expression levels. Log-rank test was used for survival analysis.

Representative images of LRIG3 IHC staining from different grade gliomas are shown in Figure 1A, while the representative images of HE, GFAP, Iba-1, CD163, and CD31 of each grade glioma are shown in Figure S1 demonstrating that LRIG3 is mainly stained on the glial cells and low grade glioma cells. LRIG3 expression was significantly higher in grade II cases than in grades III and IV cases (*p* = 0.026 and *p* = 0.00012, respectively; Figure 1B). No significant difference was found between grades III and IV cases (*p* = 0.152; Figure 1B). These data indicated that LRIG3 was highly expressed in LGGs (WHO grade II) in compared with that in HGGs (WHO grade III and IV).

Kaplan–Meier survival curves of different grade gliomas are presented in Figure 1C. Because most grade II (18/20) patients are still being followed after 30 months, indicating an increased survival rate than those of grades III and IV cases, we further estimated the association between the LRIG3 expression levels and OS of HGG patients in this study. Samples with IHC staining scores of 0–3 were considered as LRIG3-low expressed, while 4–12 were considered as LRIG3-high expressed. A generally high expression of LRIG3 in patients with grade III gliomas was correlated with good prognosis compared with that in patients with low LRIG3 expression, but the difference was not statistically significant (*p* = 0.2112; Figure 1D). In patients with grade IV glioma, higher LRIG3 expression levels were significantly associated with prolonged post-operative overall survival (*p* = 0.0464; Figure 1E). For HGG patients, high LRIG3 expression levels were significantly associated with positive prognosis (*p* = 0.0233; Figure 1F).

Collectively, these results demonstrated that LRIG3 expression levels in glioma samples were negatively associated with WHO grades, and higher LRIG3 expression levels were associated with favorable prognosis in HGG patients.

### Demonstration of sLRIG3 in the Culture Supernatants of Glioma Cells Overexpressing LRIG3

**Figure 2:**
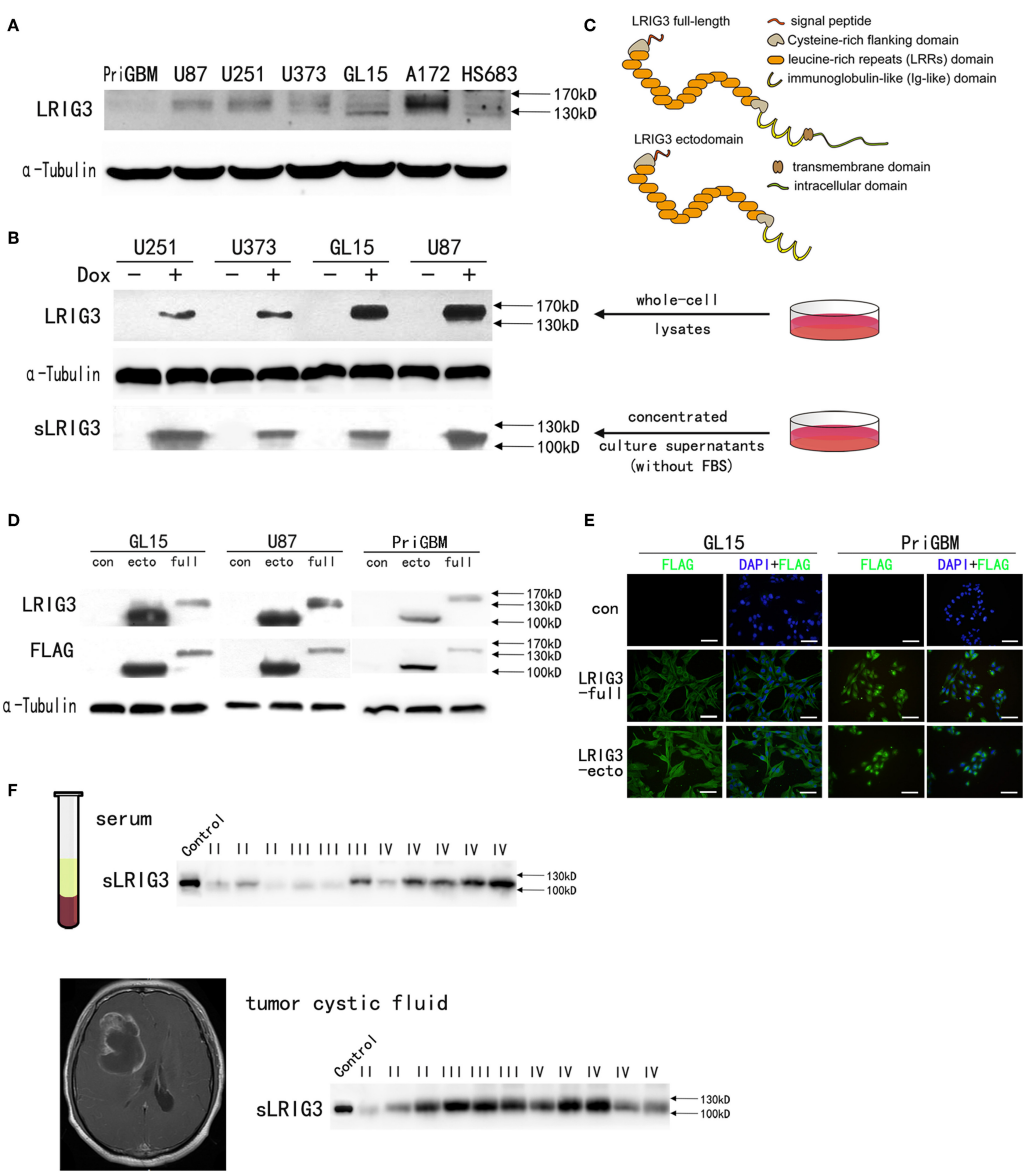
Existence of soluble LRIG3 in the cell culture supernatant, patients' serum and tumor cystic fluid. **(A)** Western blot analysis of LRIG3 expression levels in different glioma cell lines. **(B)** LRIG3 expression in whole-cell lysates of inducible LRIG3-transduced glioma cell lines and soluble LRIG3 (sLRIG3) in the concentrated conditioned medium from the corresponding cell culture, analyzed by western blotting. α-Tubulin was used as an internal control for cell lysates. **(C)** Schematic diagram of the domain organization of the full-length LRIG3 and LRIG3 ectodomain. Indicated are a signal peptide, a cysteine-rich N-flanking domain, 15 leucine-rich repeat domains, three immunoglobulin (Ig)-like domains, a transmembrane domain, and an intracellular tail. **(D)** Verification of LRIG3 (LRIG3-full) and LRIG3 ectodomain (LRIG3-ecto, sLRIG3) overexpression in GL15, U87, and PriGBM cell lines by western blot analysis. Both proteins were tagged with FLAG. **(E)** Immunofluorescence staining of LRIG3 in GL15 and PriGBM cell lines stably expressing full-length LRIG3 and LRIG3 ectodomain (LRIG3-ecto, sLRIG3), both of which were tagged with FLAG. FLAG: green; nuclei: blue (DAPI). Scale bars, 50 μm. **(F)** Western blotting of sLRIG3 in the serum and tumor cystic fluid of patients (Control represents cell lysate from GL15 cells overexpressing the LRIG3 ectodomain; I, III, and IV refer to the WHO grades of the samples).

Previous studies have shown that soluble ectodomain of LRIG1 (sLRIG1) and soluble ectodomain of LRIG2 (sLRIG2) are released into the culture supernatants and have the same functions as their corresponding full-length proteins (11, 13). To investigate whether the ectodomain of LRIG3 could be released from glioma cells, LRIG3 expression in all six glioma cell lines and one patient-derived adherent cell line, PriGBM, was examined by western blotting. The basal expression levels of LRIG3 protein were relatively low, with the exception of A172 cell line (Figure 2A). Then we constructed glioma cell lines with inducible expression of LRIG3. The upper panel of Figure 2B shows the detection of LRIG3 in whole-cell lysates of glioma cell lines transduced with inducible pLVX-TRE3G-LRIG3 expression vector, with or without the addition of Dox, while the lower panel shows the detection of sLRIG3 in the corresponding concentrated culture supernatants. Figure 2C illustrates the domain organization of full-length LRIG3 and LRIG3 ectodomain. Taken together, these results indicated the existence of sLRIG3 and that sLRIG3 could be released from glioma cells overexpressing LRIG3. As the LRIG3 antibody we used recognizes the ectodomain of LRIG3 and the molecular weight for sLRIG3 is about 110 kD, which is shorter than the full length LRIG3 in Figure 2B, sLRIG3 should be the ectodomain portion of LRIG3 protein.

### Construction of Glioma Cells Overexpressing LRIG3 and sLRIG3 as Well as Analysis of sLRIG3 in the Serum and Glioma Cystic Fluid of Patients

To investigate the functions of LRIG3 and sLRIG3, GL15, U87, and PriGBM cells were transduced with expression vectors encoding full-length LRIG3 or ectodomain of LRIG3 (sLRIG3), and empty vector control. The expression of LRIG3 (LRIG3-full) and soluble LRIG3 ectodomain (LRIG3-ecto, sLRIG3) was successfully detected using anti-LRIG3 and anti-FLAG antibodies (Figure 2D). *In situ* immunofluorescence staining showed that cells transduced with those expression vectors stably expressed FLAG (Figure 2E), which was in line with the results of western blotting.

Although it is well-known that many gliomas possess cystic lesions, the mechanism is still unknown. Researchers found that cyst fluid from cystic, malignant brain tumors provides a reservoir of nutrients for tumor cells (39); further functional research on this phenomenon has not been conducted. Patients with cystic GBMs have longer median survival time after surgery than those with noncystic GBMs (40, 41). As the microenvironment surrounding tumor cells has received considerable attention for glioma growth, and sLRIG3 has been demonstrated to exist in the culture supernatants of glioma cells, understanding whether it is presented in the glioma cystic fluid or the serum of patients with glioma required further investigation. As shown in Figure 2F, western blot analysis confirmed the existence of sLRIG3 in these samples.

Taken together, these results showed that glioma cell lines stably expressing LRIG3 and LRIG3 ectodomain were successfully established and demonstrated the existence of sLRIG3 in the serum and glioma cystic fluid of patients.

### LRIG3 and sLRIG3 Decrease the Proliferation and Anchorage-Independent Growth of Glioma Cells

**Figure 3:**
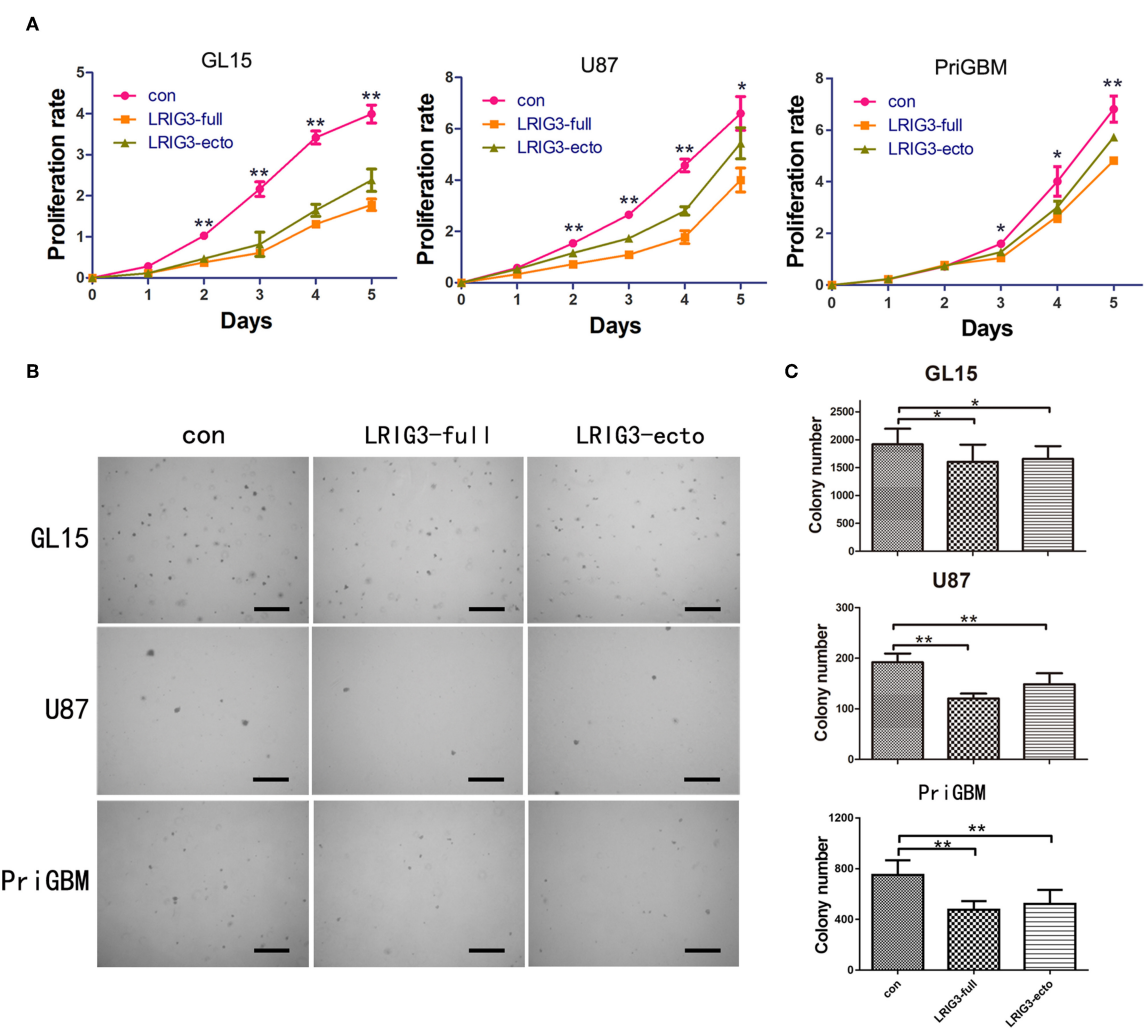
LRIG3 and sLRIG3 decrease proliferation and anchorage-independent growth of glioma cells. **(A)** Analysis of proliferation rates with CCK-8 in GL15, U87, and PriGBM cells overexpressing LRIG3 and LRIG3 ectodomain proteins compared with that in the control cells. **(B)** Anchorage-independent proliferation with colony formation assays of GL15, U87, and PriGBM cells. Representative images of soft agar colonies in each group are presented. Scale bars, 500 μm. **(C)** Quantitative analysis of colony numbers per well of each group (Data represent the mean ± SD of three independent experiments. ^*^p < 0.05; ^**^p < 0.01 vs. control group; one-way ANOVA).

Given that sLRIG3 is released from glioma cells overexpressing LRIG3 and can be detected in serum and glioma cystic fluid samples of patients, we further investigated the functions of LRIG3 and sLRIG3 in gliomas. The CCK-8 assay demonstrated that both LRIG3 and sLRIG3 significantly decreased the proliferation rates of glioma cells as compared with that in the control group (Figure 3A). Furthermore, soft agar colony formation assays showed that significantly fewer and smaller colonies were formed within the experimental groups than those in the control group (Figures 3B,C), indicating that overexpression of LRIG3 and sLRIG3 decreased the anchorage-independent growth of glioma cells. Taken together, these results demonstrated that both LRIG3 and sLRIG3 effectively decrease the growth and tumor colony formation of glioma cells *in vitro*.

### LRIG3 and sLRIG3 Inhibit the Invasion and Migration Capabilities of Glioma Cells

**Figure 4:**
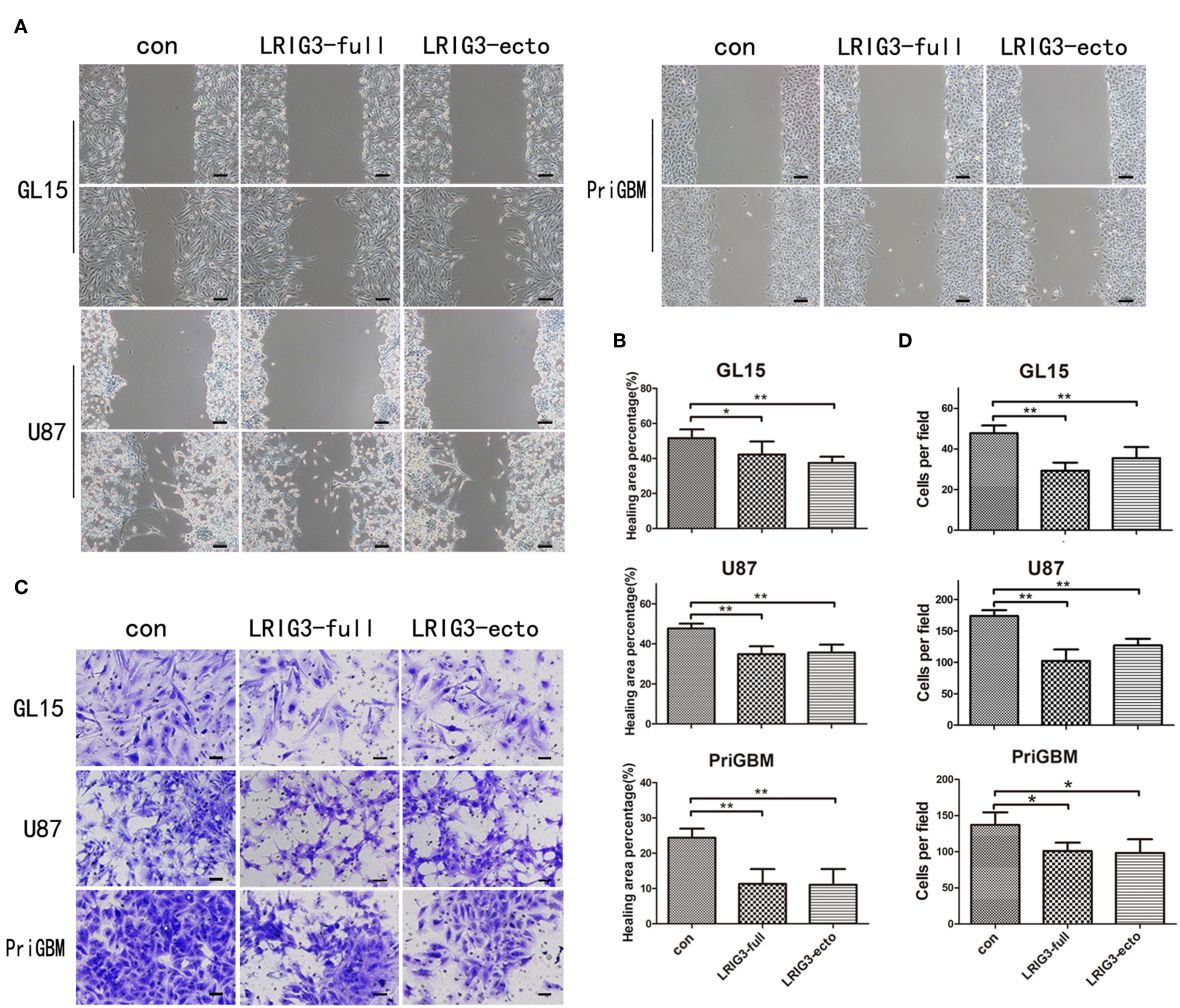
LRIG3 and sLRIG3 inhibit migration and invasion of glioma cells. **(A)** Wound-healing assay of GL15, U87, and PriGBM cells overexpressing full-length LRIG3 and LRIG3 ectodomain proteins (sLRIG3). Representative images of wounded cell monolayers. Scale bar, 100 μm. **(B)** Quantification of healing areas of the different groups from each cell lines (Data represent the mean ± SD of triplicates from healing areas of one experiment. *p < 0.05; **p < 0.01 vs. control group; one-way ANOVA). **(C)** Invasion capacity as measured by transwell invasion assays. Representative images of the microscopic fields are shown. Scale bar, 50 μm. **(D)** Numbers of migrated cells per microscopic field were analyzed from five predetermined fields (Data represent the mean ± SD. *p < 0.05; **p < 0.01 vs. control group; one-way ANOVA).

In addition to rapid proliferation, glioma cells exhibit a highly invasive nature, which often leads to incomplete neurosurgical resection, resulting in recurrence and malignant progression (42). Thus, we investigated whether LRIG3 and sLRIG3 could affect the invasion and migration capabilities of glioma cells by performing the wound-healing assay and Matrigel invasion assay. LRIG3- and sLRIG3-overexpressing cells healed significantly less areas 24 h after wounding (Figures 4A,B). Similarly, Matrigel invasion assays showed that LRIG3 and sLRIG3 overexpression significantly decreased the average number of invaded cells (Figures 4C,D). These assays demonstrated that LRIG3 and sLRIG3 attenuated the migration and invasion of glioma cells.

### LRIG3 and sLRIG3 Inhibit the Phosphorylation of MET and the Components of the Downstream PI3K/Akt/mTOR Pathway

**Figure 5:**
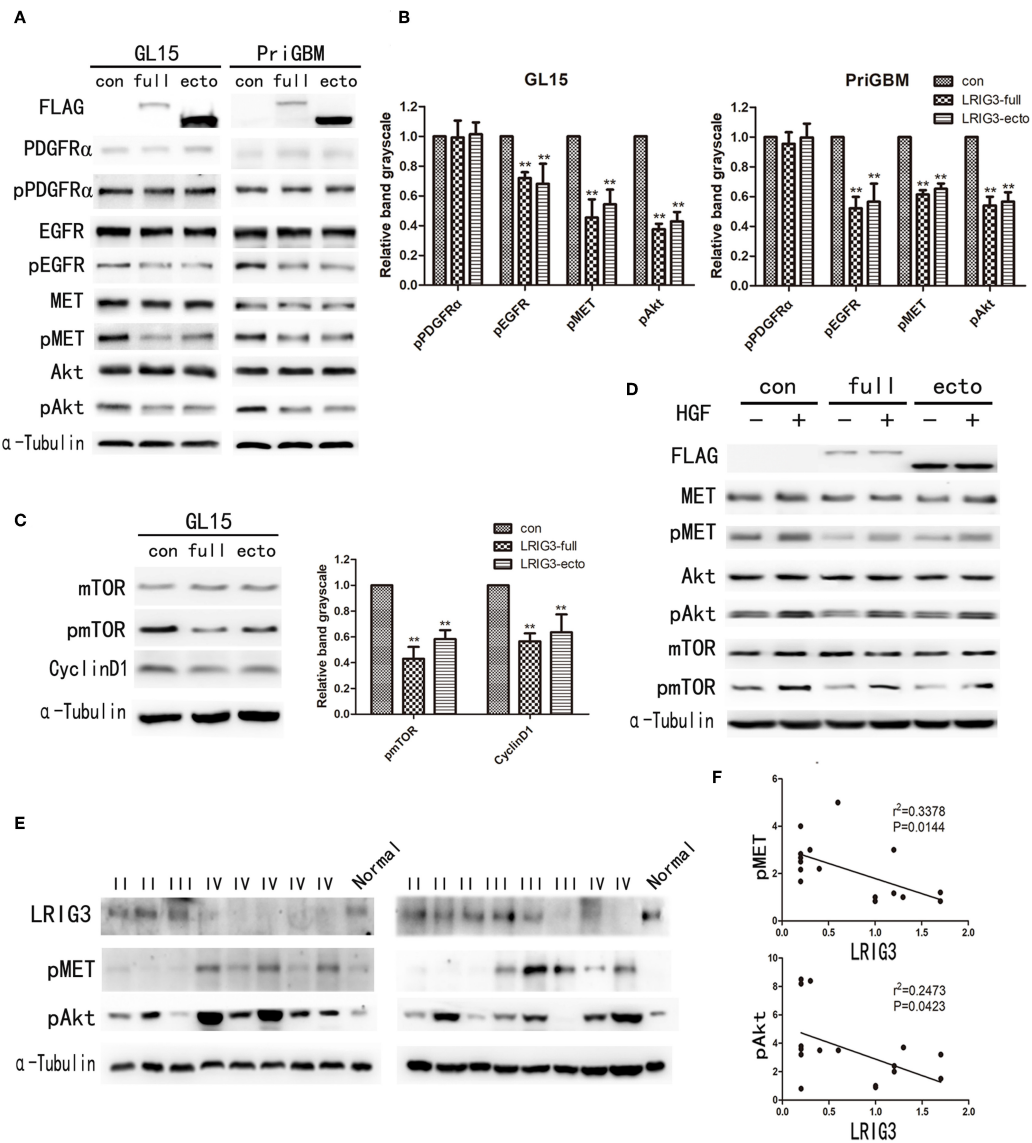
LRIG3 and sLRIG3 inhibit the phosphorylation of MET and components of the downstream PI3K/Akt pathway. **(A)** Western blot analysis of cell lysates from GL15 and PriGBM cells overexpressing full-length LRIG3 and LRIG3 ectodomain proteins (sLRIG3) for FLAG, PDGFRα/pPDGFRα, EGFR/pEGFR, MET/pMET, and Akt/pAkt. α-Tubulin was used for loading control. **(B)** Analysis of quantification of the bands intensity of pPDGFRα, pEGFR, pMET, and pAkt from three repeated experiments (^**^p < 0.01 vs. control group; one-way ANOVA). **(C)** Immunoblot analysis of mTOR/pmTOR and cyclin D1 in GL15 cells. α-Tubulin was used for loading control. Analysis of quantification of the bands intensity was shown in the right panel (^**^p < 0.01 vs. control group; one-way ANOVA). **(D)** GL15 cells with different LRIG3 expression status treated with rhHGF (20 ng/ml) for 15 min. Western blot analysis was carried out for FLAG, MET/pMET, Akt/pAkt, and mTOR/pmTOR. **(E)** Immunoblot analysis of LRIG3, pMET, and pAkt in 16 glioma tissue samples. Normal control represents one normal frontal brain tissue obtained from decompression surgery of a patient with traumatic brain injury. **(F)** Correlation analysis of the band intensity of pMET and pAkt with LRIG3 in the 16 glioma specimens based on densitometry of immunoblots from **(E)** (*p*-values were determined by Pearson correlation analysis; *r* refers to the correlation coefficient).

According to the Cancer Genome Atlas (TCGA) genetic screening, 86% of human glioblastoma samples harbor at least one genetic alteration event in the core RTK pathways, and EGFR, PDGFRα, and MET are most commonly amplified RTKs in glioblastomas (20, 21). To investigate regulation mechanisms of LRIG3 and sLRIG3 relating to the malignancy of glioma cells, western blot analysis was performed to evaluate expression and activation levels of PDGFRα, EGFR, MET, Akt, and mTOR. Phosphorylation of EGFR was inhibited (Figures 5A,B), which was consistent with our previous study results (19). In contrast, EGFR levels were not influenced by different LRIG3 protein levels (Figures 5A,B), which is inconsistent with the study by Rafidi et al. (17). Although phosphorylation of PDGFRα was not affected by the status of LRIG3 in GL15 and PriGBM cells, MET and Akt phosphorylation was inhibited by overexpression of LRIG3 and sLRIG3 compared with that in the control group (Figures 5A,B). Further downstream signaling molecular analysis showed that the phosphorylation of mTOR was consistent with the phosphorylation of MET and Akt in GL15 cells (Figure 5C). Additionally, cyclin D1 expression was decreased in cells overexpressing LRIG3 and sLRIG3 (Figure 5C).

The human MET proto-oncogene is located in the 7q31 locus of chromosome 7, and HGF is located on chromosome 7q21.1 (43). Analysis of TCGA data showed that ~30% of GBMs display overexpression of HGF and MET, suggesting instances in the patient population where autocrine HGF activation occurs (44). HGF/MET signaling has been suggested to be important for cell survival, angiogenesis, and invasion in glioblastoma (25, 26). After treatment with the MET agonist rhHGF (20 ng/ml) for 15 min, rhHGF-stimulated activation of MET and its downstream components were also inhibited by LRIG3 and sLRIG3 (Figure 5D). This observation further supported that LRIG3 and sLRIG3 decreased phosphorylation of Akt and mTOR through the MET signaling pathway.

Furthermore, we assessed LRIG3 expression levels in 16 fresh frozen glioma tissues (5 grade II, 4 grade III, and 7 glioblastoma samples) and one normal brain tissue via western blotting (Figure 5E). The results revealed that phosphorylation levels of MET and Akt were negatively correlated with LRIG3 expression levels (*n* = 16; *p* = 0.0144 and 0.0423, respectively; Figure 5F). These findings are consistent with the results that LRIG3 inhibited the phosphorylation of the components of the MET/PI3K/Akt pathway in glioma cell lines.

### Knockdown of LRIG3 Promotes Proliferation and Migration of Glioma Cells *in vitro*

**Figure 6:**
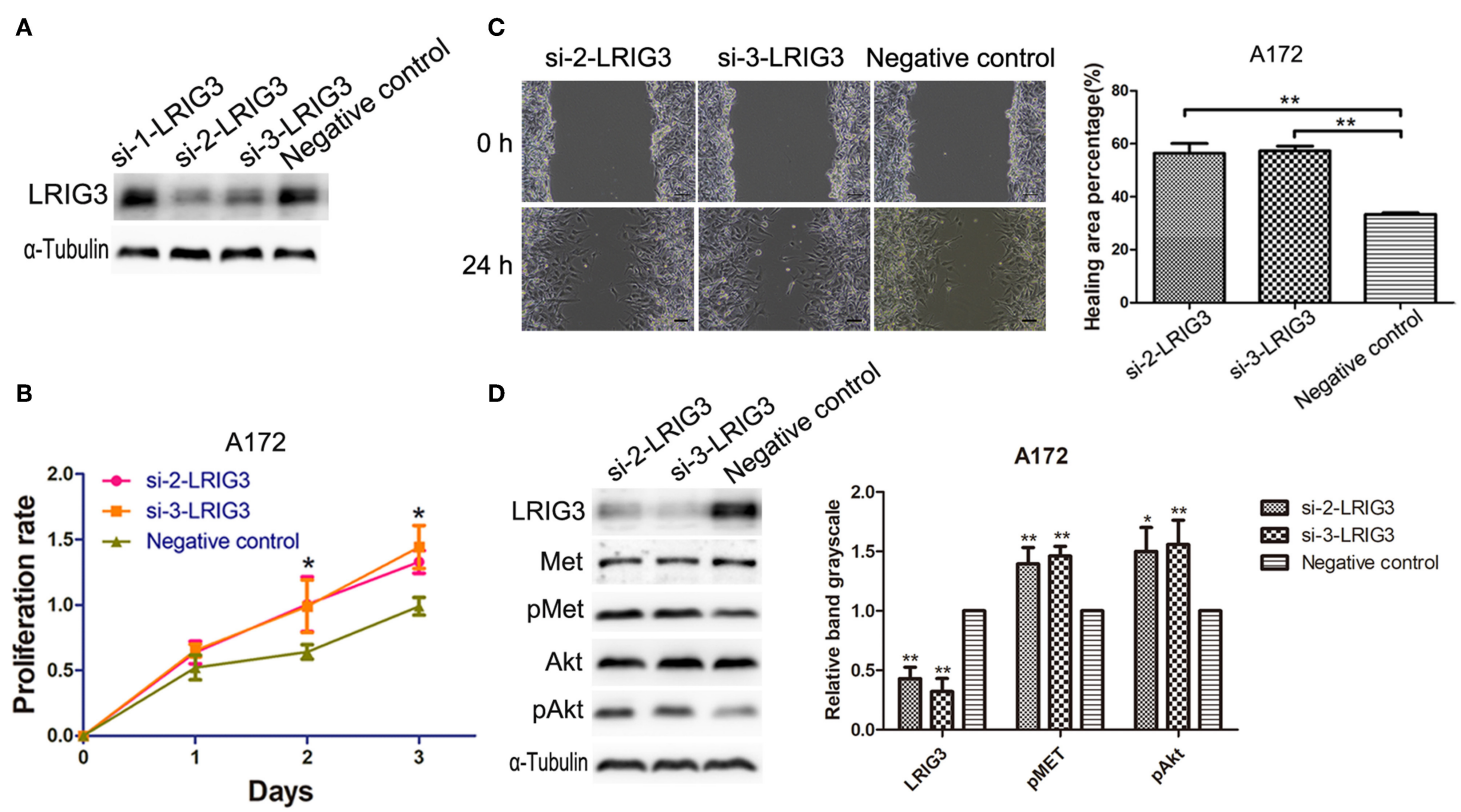
Downregulation of LRIG3 promotes proliferation and migration of glioma cells. **(A)** Silencing efficiency of LRIG3 knockdown in A172 cells through siRNA, detected by western blotting. **(B)** Effects of LRIG3 knockdown on cell growth *in vitro*, as evaluated by CCK-8 assays. **(C)** Scratch assays were used to measure the percentage of healing areas. Scale bar, 100 μm. **(D)** Western blot analysis of the effects of LRIG3 knockdown on the activation of MET and Akt (Data represent the mean ± SD of three repeated experiments; ^*^p < 0.05; ^**^p < 0.01 vs. negative control; one-way ANOVA).

To further underscore the functions of LRIG3 on glioma cells, we generated LRIG3-knockdown cell line, A172. After demonstrating that si-2-LRIG3 and si-3-LRIG3 effectively silenced LRIG3 expression in A172 cell lines (Figure 6A), growth curves and migration assays indicated that knockdown of LRIG3 protein facilitated the proliferation and migration of A172 cells (Figures 6B,C). Furthermore, western blotting showed enhanced phosphorylation levels of MET and Akt after LRIG3 knockdown (Figure 6D). Consequently, downregulation of LRIG3 enhanced the proliferation and migration of glioma cells.

### LRIG3 and sLRIG3 Decrease Tumorigenesis *in vivo*

**Figure 7:**
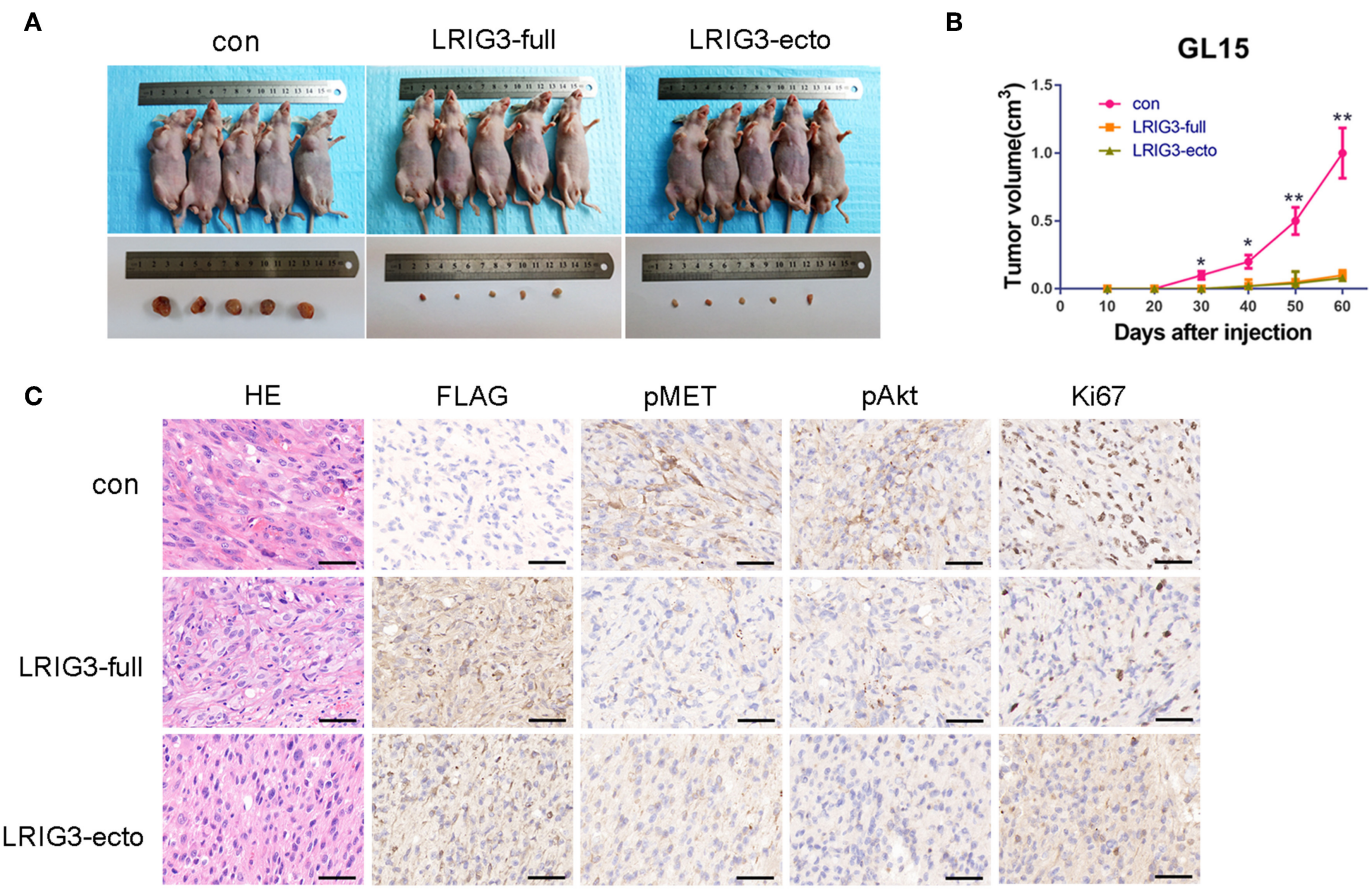
LRIG3 and sLRIG3 decrease tumorigenesis of glioma cells *in vivo*. **(A)** Images of nude mice bearing GL15 xenografts overexpressing LRIG3 and LRIG3 ectodomain (sLRIG3) and the control GL15 xenografts on day 60 after implantation. The harvested tumors were presented in the lower panel. **(B)** Tumor growth curves were plotted according to the monitored tumor size every 10 days for 60 days. Data represent the mean ± SD; *n* = 5; ^*^p < 0.05, ^**^p < 0.01; one-way ANOVA. **(C)** IHC staining of FLAG, pMET, pAkt, and Ki67 in transplanted tumors. Representative photographs for each antibody and each group are shown. Scale bars, 50 μm.

To confirm whether overexpression of LRIG3 and sLRIG3 could affect tumor growth *in vivo*, LRIG3 and sLRIG3 overexpression and control cells of GL15 were injected into nude mice as described. Tumor-burdened nude mice and the harvested tumors are shown in Figure 7A. Figure 7B demonstrated that the tumors in the experimental groups overexpressing LRIG3 and sLRIG3 grew slower than those in the control group. These results indicate that LRIG3 and sLRIG3 ameliorate growth and tumorigenesis of glioma cells *in vivo*.

The tumor xenografts of GL15 cells were also subjected to IHC staining. As shown in Figure 7C, the phosphorylation levels of MET and Akt were lower in tumors overexpressing LRIG3 and sLRIG3 than in the control group. Expression of Ki67, a marker of cell proliferation activity, was dramatically decreased in the experimental groups compared with that in the control group (Figure 7C). These results demonstrated that LRIG3 and sLRIG3 overexpression decreased tumor xenograft growth through the same mechanism observed in the *in vitro* experiments.

### Serum sLRIG3 Is a Potential Prognostic Marker for HGG Patients

**Figure 8:**
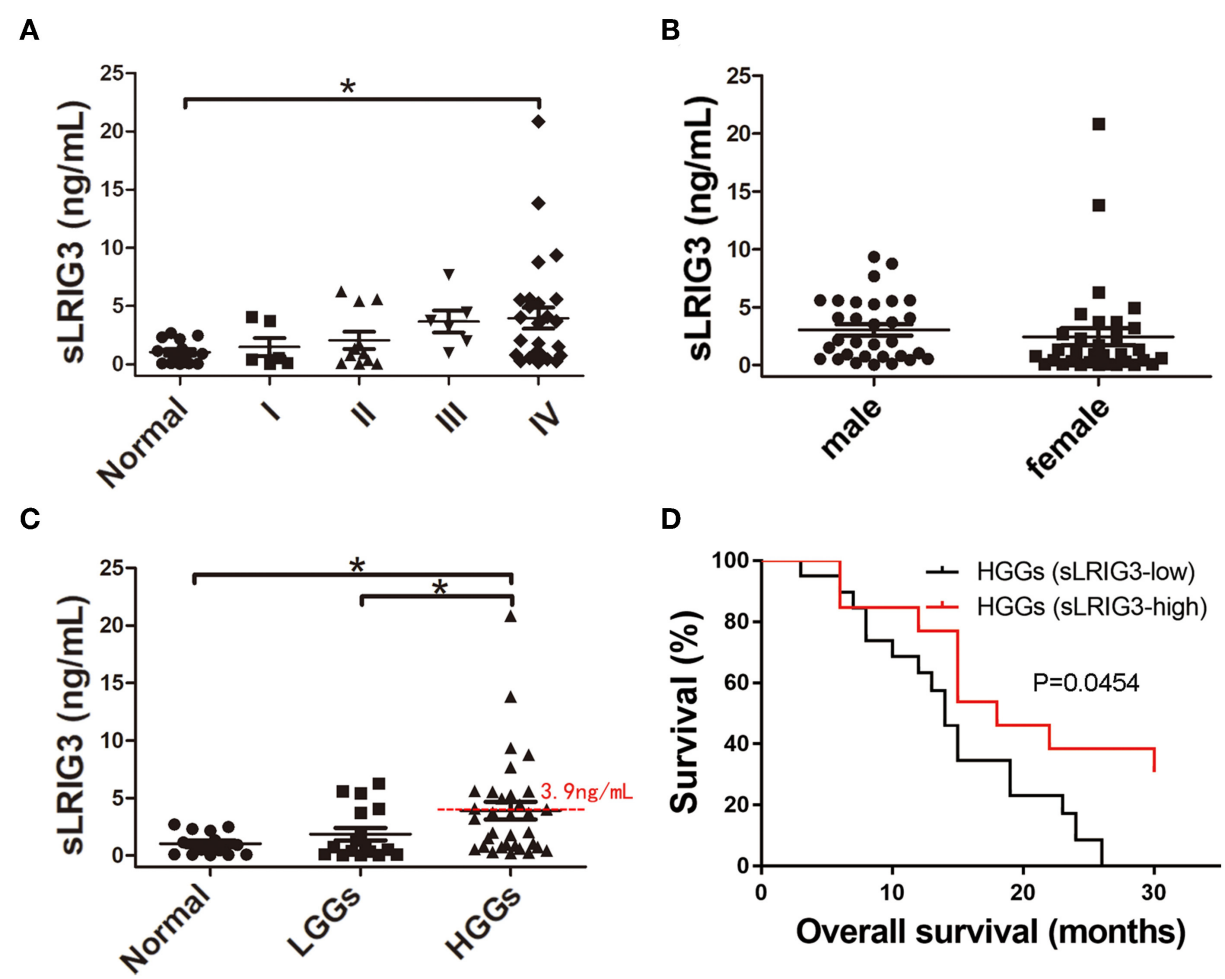
Correlation between the serum sLRIG3 levels and the prognosis of patients with HGG. **(A)** Serum sLRIG3 levels of healthy people and glioma patients with different WHO grades (Data represent the mean ± SEM; ^*^p < 0.05; one-way ANOVA). **(B)** Serum sLRIG3 levels of patients with different sex (31 males and 33 females; Data represent the mean ± SEM; *p* = 0.504; Student's *t*-test). **(C)** Serum sLRIG3 levels of the groups from LGG and HGG patients. **(D)** Kaplan–Meier overall survival curves of patients with HGG (*n* = 33), divided according to the serum sLRIG3 threshold level of 3.9 ng/ml. Log-rank test was used for survival analysis, and censored data are indicated by vertical lines.

Our aforementioned study demonstrated that sLRIG3 existed in serum samples from glioma patients through western blotting, and overexpression of sLRIG3 inhibited the proliferation and invasion of glioma cells. We hypothesized that serum sLRIG3 levels might be useful for providing prognostic information for glioma patients. To evaluate the circulating sLRIG3 levels in glioma patients, a total of 64 serum samples underwent ELISA analysis, which included serum samples from patients with gliomas of all grades (*n* = 50; 6 grade I, 11 grade II, 6 grade III, and 27 grade IV cases) and healthy controls (*n* = 14). The mean levels of sLRIG3 in serum samples appeared to be positively associated with WHO grades; however, only grade IV cases showed statistically significant differences compared with that of the control group (*p* = 0.012; Figure 8A). Although the two sample with the highest sLRIG3 levels are from female glioblastoma patients, serum sLRIG3 levels showed no significant correlation with sex, presented in Figure 8B (*p* = 0.504). Furthermore, sLRIG3 levels showed imparity between HGGs and LGGs (HGGs vs. LGGs, *p* = 0.048; HGGs vs. normal, *p* = 0.01; Figure 8C). Serum sLRIG3 levels ranged from 0 to 21 ng/ml in HGG group and were further grouped according to their sLRIG3 levels: the high sLRIG3 group included samples with levels higher than the mean level of 3.9 ng/ml, whereas the rest of the samples were classified in the low sLRIG3 group. Survival analysis demonstrated that higher serum sLRIG3 levels were associated with better overall survival in HGGs (Figure 8D). Collectively, these results showed that serum sLRIG3 levels were comparatively higher in patients with HGGs than that with LGGs, and higher sLRIG3 levels were associated with better overall survival among patients with HGGs.

## Discussion

In the present study, we aimed to investigate the existence of sLRIG3 and to further our understanding of the functions of LRIG3 and sLRIG3 in glioblastoma. For the first time, we revealed that sLRIG3 was presented in the culture supernatants, patient serum and tumor cystic fluid. Further study demonstrated that both LRIG3 and sLRIG3 inhibit the proliferation, migration and invasion of glioma cells by decreasing phosphorylation of MET and downstream signaling components *in vitro* and *in vivo*. LRIG3 expression levels in the tumor samples were inversely correlated with WHO grades, whereas serum sLRIG3 levels were not the same, and further survival analysis of HGG patients revealed higher LRIG3 expression levels in tumor samples or higher serum sLRIG3 levels were associated with better overall survival. These findings support the use of LRIG3 as a prognostic marker for glioblastoma.

Previous studies have shown that growth factor pathways are constitutively activated in malignant gliomas through gene mutations and overexpression or genetic amplification of growth factor receptor genes (20). Unfortunately, several inhibitors for these receptors, showing significant antitumor activity *in vitro*, were found inactive in clinical trials (3, 4). Thus, endogenous regulation should be evaluated in further studies. In our study, both LRIG3 and sLRIG3 proteins attenuated the progression of malignant glioma through downregulating the activation of MET/PI3K/Akt/mTOR pathway *in vitro* and *in vivo*. The PI3K/Akt pathway is characterized as a critical regulator of tumor cell metabolism, growth, proliferation, and survival, and the downstream molecule of mTOR has an array of biological functions that promote hypoxic adaption and protein translation. We revealed for the first time a negative association between LRIG3/sLRIG3 and MET signaling in human glioblastoma and provided compelling evidence in support of the hypothesis that LRIG3 and sLRIG3 could serve as tumor suppressors in human gliomas.

Notably, based on our current and previous study (19), we can conclude that LRIG3 exerts no effects on EGFR and MET protein expression levels, but does regulate the phosphorylation of MET and EGFR signaling pathway components to inhibit the progression of glioblastoma. However, Rafidi et al. reported that LRIG3 stabilized and increased the expression of ErbB receptors in HEK293T cells (17), which is distinct from the results of our present study. This discrepancy may be explained by the fact that the experiments by Rafidi et al. (17) were carried out in HEK293T cells, rather than in glioma cells. Thus, we hypothesize that the regulation of LRIG3 on the RTKs might be dependent on different genetic profiles. Although we have demonstrated that LRIG3 and sLRIG3 decreased the phosphorylation of MET and downstream signaling pathway components, it remains unclear how LRIG3 reduced the phosphorylation levels of MET but not the total expression of MET, and thus further studies are required.

One histological feature of glioblastoma is microvascular proliferation, which is required for its malignant growth. Bevacizumab, an antibody targeting vascular endothelial growth factor (VEGF), is approved and comparatively widely used for the treatment of recurrent glioblastoma and combined with other therapies (45, 46). However, this agent only prolongs progression-free survival and does not affect overall survival, due to aberrant MET expression and activation, which may contribute to bevacizumab resistance (28, 47). Of note, MET and its ligand hepatocyte growth factor (HGF) also play critical roles in the proliferation, survival, migration, invasion, angiogenesis, stem cell characteristics, therapeutic resistance, and recurrence of glioblastomas (25–30). Although there are many preclinical and clinical studies about small molecular inhibitors or antibodies against MET in glioblastoma, their usefulness is limited. A phase II study demonstrated there was no evidence of further clinical benefit with the addition of onartuzumab (an antibody against MET) to bevacizumab compared with bevacizumab plus placebo in patients with recurrent glioblastoma (48). Cabozantinib (XL184), a potent inhibitor of MET and VEGFR2, showed only modest clinical activity in patients with progressive glioblastoma (49). Additionally, coexpression of MET and its ligand HGF occur frequently in cancers, including glioblastoma, and have been associated with increased malignancy and decreased patient survival (31, 32, 44). Because both LRIG3 and sLRIG3 inhibit the activation and rhHGF-induced activation of the MET signaling pathway, these proteins may be useful in combination with bevacizumab treatment to work alongside conventional anti-glioblastoma therapies to improve patient survival.

As the microenvironment surrounding tumor cells is rather important for glioma growth, and the studies about cystic glioblastoma demonstrate that patients with cystic GBMs had longer median survival time after surgery than those with noncystic GBMs (40, 41), the sLRIG3 in the glioma cystic fluid might contribute to the improved prognosis. Since sLRIG3 simultaneously exists in the peripheral blood (serum) and central tumor microenvironment (tumor cystic fluid), and higher serum sLRIG3 levels in HGG patients were associated with better overall survival, we hypothesize that sLRIG3 may possess therapeutic potential against glioblastoma. Our *in vitro* and *in vivo* studies in nude mice indicated that sLRIG3 ameliorated the malignancy of gliomas. Therefore, if sLRIG3 is shown to penetrate the blood brain barrier, it might be used as a direct therapeutic agent.

In summation, we verified for the first time that sLRIG3 has the capability of being released from glioma cells and exists in the patient serum and glioma cystic fluid, and both LRIG3 and sLRIG3 significantly inhibit the proliferation and invasion capabilities of glioma cells both *in vitro* and *in vivo* through inhibiting MET/PI3K/Akt pathway. The specific mechanisms behind LRIG3 ectodomain release from glioma cells and the pattern of the interaction with MET deserve more investigation in the future. Considering that both LRIG3 in the tumor sample and sLRIG3 in the serum samples are associated with better prognosis of HGG patients, they might represent critical prognostic markers and offer potential in tailoring treatments for patients with glioblastoma in the future.

## Conclusions

Glioblastoma is a highly malignant brain tumor, and patients exhibit a uniformly dismal prognosis with the current therapies. Previous analysis of glioma samples showed that aberrant regulations of RTK/PI3K pathway played important roles in the events of tumor formation, progression and therapeutic resistance. In this study, we demonstrated the existence of soluble ectodomain of the human LRIG3, and reported that LRIG3 and its soluble ectodomain, sLRIG3, inhibited glioma progression through the regulation of the MET/PI3K/Akt pathway both *in vitro* and *in vivo*. Clinical studies showed that LRIG3 protein expression levels in glioma samples were negatively correlated with WHO grades. For patients with HGGs, both higher LRIG3 protein expression levels in glioma tissues and higher serum sLRIG3 levels indicated improved prognosis. In summary, LRIG3 and sLRIG3 may represent critical prognostic markers in clinical practice, and sLRIG3 has potential as a therapeutic agent for glioblastoma.

## Data Availability

The raw data supporting the conclusions of this manuscript will be made available by the authors, without undue reservation, to any qualified researcher.

## Author Contributions

DG, BW, and QD discussed the project and designed the research. FC conducted the research, analyzed, interpreted the data, and contributed to writing the manuscript. PZ and MD performed part of the animal experiment. YL performed part of the cell experiment. FC and PZ contributed to the follow-up records of patients and analysis. FM, YH, and HW discussed the project, analyzed, and interpreted the data. DK and QX provided research material, discussed the project, and analyzed the IHC staining results. BW and DG analyzed and revised the manuscript. All authors read and approved the final manuscript.

### Conflict of Interest Statement

The authors declare that the research was conducted in the absence of any commercial or financial relationships that could be construed as a potential conflict of interest.

## Supplementary Material

Figure S1 Representative images of series continuous slices from different grade gliomas with the HE staining and IHC staining of GFAP, Iba-1, CD163, and CD31. GFAP, glial fibrillary acidic protein, is a marker of astrocyte; Iba-1 is a marker for Microglia cells; CD163 is a marker for mononuclear macrophages; CD31 is a marker of vascular endothelial cells. All these pictures revealed that it is not the resident macrophages, myeloid cells or other stromal cells like vascular endothelial cells which expressed LRIG3. Scale bar, 100 μm.

Table S1 Characteristics of patients with tumor samples for western blotting.

Table S2 Characteristics of glioma patients possessing cystic lesions.

## Abbreviations

BME: basal medium Eagle
EGFR: epidermal growth factor receptor
FBS: fetal bovine serum
IHC: immunohistochemistry
LRIG: the human leucine-rich repeats and immunoglobulin-like domains
MET: mesenchymal-epithelial transition factor
mTOR: mammalian target of rapamycin
PDGFRα: platelet derived growth factor receptor α
rhHGF: recombinant human hepatocyte growth factor
RTK: receptor tyrosine kinase
WHO: World Health Organization.
