# Corrigendum: The Prognostic and Therapeutic Potential of LRIG3 and Soluble LRIG3 in Glioblastoma

**DOI:** 10.3389/fonc.2020.591112

**Published:** 2020-09-25

**Authors:** Fangling Cheng, Po Zhang, Qungen Xiao, Youwei Li, Minhai Dong, Heping Wang, Dong Kuang, Yue He, Qiuhong Duan, Feng Mao, Baofeng Wang, Dongsheng Guo

**Affiliations:** ^1^Department of Neurosurgery, Tongji Hospital, Tongji Medical College, Huazhong University of Science and Technology, Wuhan, China; ^2^Chinese-German Lab of Molecular Neuro-oncology of Tongji Hospital, Huazhong University of Science and Technology, Wuhan, China; ^3^Institute of Pathology, Tongji Hospital, Tongji Medical College, Huazhong University of Science and Technology, Wuhan, China; ^4^Department of Pathology, School of Basic Medicine, Tongji Medical College, Huazhong University of Science and Technology, Wuhan, China; ^5^Department of Biochemistry and Molecular Biology, School of Basic Medicine, Huazhong University of Science and Technology, Wuhan, China

**Keywords:** glioma, glioblastoma, LRIG3, soluble LRIG3, MET/PI3K/Akt pathway, prognosis

In the original article, there was a mistake in Figure 4 as published. The second and third image in the first row of Figure 4A are the same. The corrected Figure 4 appears below.

**Figure 4:**
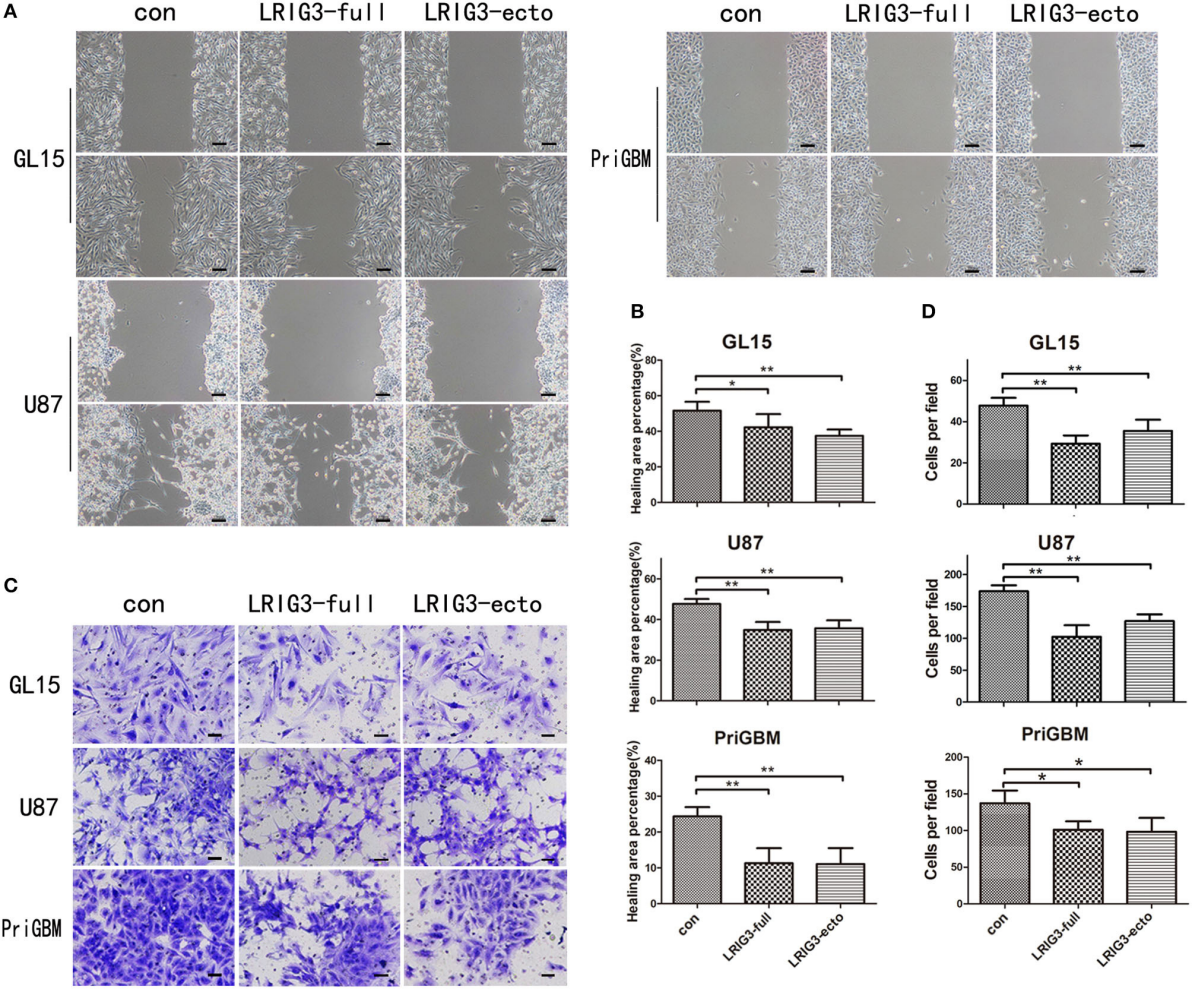
LRIG3 and sLRIG3 inhibit migration and invasion of glioma cells. **(A)** Wound-healing assay of GL15, U87, and PriGBM cells overexpressing full-length LRIG3 and LRIG3 ectodomain proteins (sLRIG3). Representative images of wounded cell monolayers. Scale bar, 100 μm. **(B)** Quantification of healing areas of the different groups from each cell lines (Data represent the mean ± SD of triplicates from healing areas of one experiment. *p < 0.05; **p < 0.01 vs. control group; one-way ANOVA). **(C)** Invasion capacity as measured by transwell invasion assays. Representative images of the microscopic fields are shown. Scale bar, 50 μm. **(D)** Numbers of migrated cells per microscopic field were analyzed from five predetermined fields (Data represent the mean ± SD. *p < 0.05; **p < 0.01 vs. control group; one-way ANOVA).

The authors apologize for this error and state that this does not change the scientific conclusions of the article in any way. The original article has been updated.

