# Supplementary figures and images for: The Prognostic and Therapeutic Potential of LRIG3 and Soluble LRIG3 in Glioblastoma
Source: Front Oncol. 2019 Jun 6;9:447. doi: 10.3389/fonc.2019.00447 (PMC6563081; doi:10.3389/fonc.2019.00447)

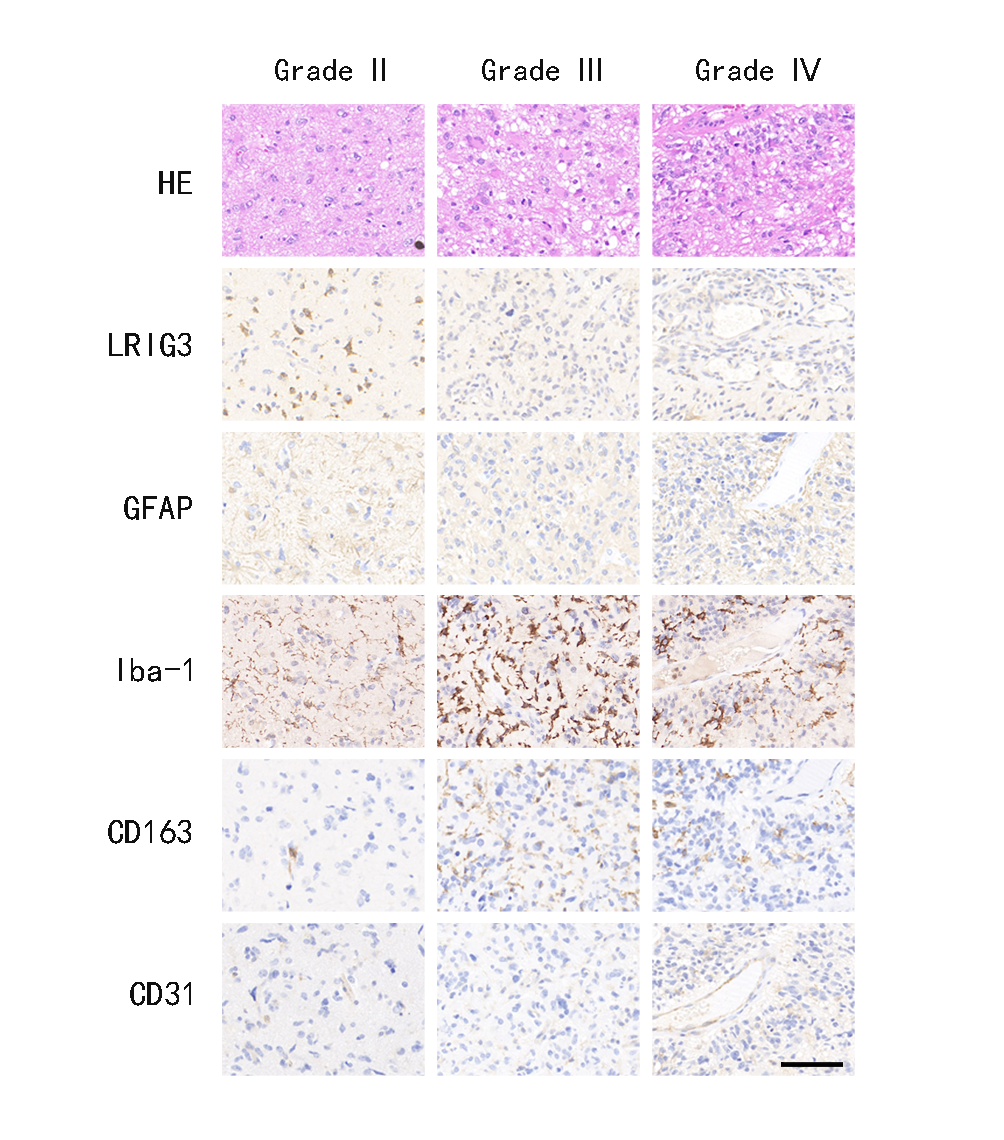

Supplement: Figure S1 — Representative images of series continuous slices from different grade gliomas with the HE staining and IHC staining of GFAP, Iba-1, CD163, and CD31. GFAP, glial fibrillary acidic protein, is a marker of astrocyte; Iba-1 is a marker for Microglia cells; CD163 is a marker for mononuclear macrophages; CD31 is a marker of vascular endothelial cells. All these pictures revealed that it is not the resident macrophages, myeloid cells or other stromal cells like vascular endothelial cells which expressed LRIG3. Scale bar, 100 μm. [file Image_1.tif]
